# Experiences of burnout, anxiety, and empathy among health profession students in Qatar University during the COVID-19 pandemic: a cross-sectional study

**DOI:** 10.1186/s40359-023-01132-3

**Published:** 2023-04-13

**Authors:** Ruba Sulaiman, Sara Ismail, Mujahed Shraim, Maguy Saffouh El Hajj, Tanya Kane, Alla El-Awaisi

**Affiliations:** 1 Department of Clinical Pharmacy and Practice, College of Pharmacy, QU Health, Qatar University, Doha, Qatar; 2 Department of Public Health, College of Health Sciences, QU Health, Qatar University, Doha, Qatar; 3 Department of Population Medicine, College of Medicine, QU Health, Qatar University, Doha, Qatar

**Keywords:** Burnout, Anxiety, Empathy, Health Profession Students, COVID-19, Qatar

## Abstract

**Background:**

The prevalence of burnout and anxiety is constantly increasing among health profession students worldwide. This study evaluates the prevalence of burnout and its relationship to anxiety and empathy during the COVID-19 pandemic among health profession students in the main governmental institution in Doha, Qatar using validated instruments.

**Methods:**

A cross-sectional survey of health profession students using validated instruments was employed. The Maslach Burnout Inventory-General Students Survey (MBI-GS(S)) to measure burnout; The Generalized Anxiety Disorder (GAD-7) to measure anxiety; and Interpersonal Reactivity Index (IRI) to measure empathy were utilized. Descriptive statistics and multivariable linear regression were used.

**Results:**

Of the 1268 eligible students, 272 (21.5%) completed the online survey. Burnout was found to be prevalent amongst the students. The mean scores for the MBI-GS(S) subscales of emotional exhaustion, cynicism, and professional efficacy were 4.07, 2.63, and 3.97, respectively. Anxiety was found to be a strong predictor for burnout and burnout was positively associated with empathy.

**Conclusions:**

Findings from this study demonstrated relationships between health profession students’ burnout, anxiety, and empathy. These findings might have an impact on the development of curriculum interventions to enhance student well-being. More burnout awareness and management programs that cater to the specific needs of health profession students are needed. Furthermore, findings of this study may have implications for future educational interventions during times of crisis or how this can be used to improve student experiences in normal times.

## Introduction

Burnout has been characterised as a psychological syndrome and a state of continuous stress [1]. It involves high emotional exhaustion, depersonalization, and low sense of personal accomplishment [1, 2]. Initially, burnout was connected exclusively with occupations and professions that require constant and direct contact with people, however burnout has recently been found to affect students to the same extent due to university stressors such as high academic demands, deadlines, balancing academic and social life, and financial pressures [3, 4]. The causes of student burnout are multidimensional, including exhaustion from academic demands and stressors, a sceptical attitude towards their own education, and feeling incompetent and ineffective [5]. Burnout assessment comprises three subscales: emotional exhaustion, cynicism, and professional inefficiency [6]. Intrapersonal characteristics such as student personality and interpersonal factors, such as social support have been linked to burnout amongst students [7].

Generally, burnout is a growing concern among health profession students and can have negative impact on their mental health, professionalism, and empathy due to the high demand of the different medical and health education which can be long lasting [3, 8–11]. It can also affect interprofessional relationship with other health professionals and patients [11]. Experiencing burnout, for example, can depersonalize health professional students and reduce their empathy for patients [12–15] and as a result they may exhibit unprofessional behaviors [10], leading to a reduction in the quality of provided care [16]. Several studies have elaborated that empathy is a crucial trait for health professionals as it strengthens the trust and relationship between the providers and their patients [17], as well as it leads to higher patient satisfaction and adherence to their therapeutic regimens which will increase the positive outcomes of therapy [18]. Also, empathy benefits the health professionals by protecting them from burnout, distress and improving their communication skills [18]. Many studies have shown that elevated levels of burnout are associated with lower levels of empathy [12, 13, 19, 20]. Additionally, a recent study on nursing students in Brazil showed that there are negative correlations between empathy (empathic concern and perspective-taking) and depersonalization, self-efficacy, and emotional exhaustion, as well as positive correlations between personal accomplishment, empathy and self-efficacy [20].

Furthermore, the emotional exhaustion and cynicism subscales of burnout have been shown to positively relate to anxiety symptoms, meaning that anxiety can be a predictor of burnout in some students [21]. Anxiety is often accompanied by unspecified psychological and physical symptoms like palpitations, paleness, and shortness of breath [22]. Furthermore, a study conducted on medical students in Nepal showed that depression, anxiety, being a first-year resident and limited participation in extracurricular events increased their risk of developing burnout [23]. Moreover, a meta-analysis demonstrated that the prevalence of anxiety in medical students globally was 33.8%, which may negatively affect their quality of life and the quality of care they provide [24].

When the global COVID-19 pandemic commenced, everyone was affected, including university students who were expected to quickly adapt to online learning and to study in isolation of their peers. University students were particularly vulnerable to the impact COVID-19 on their life, academic performance andsense of belonging and socioeconomic status [25–27]. Furthermore, the sudden shift to online learning under quarantine have undoubtedly increased the risk of burnout and physiological distress which could negatively influenced student academic performance mental and physical well-being. In terms of the impact on health profession students, a study conducted on medical students across the United States of America (USA) reported that the pandemic prevented the students from participating in clinical rotations and increased their stress and anxiety levels [28]. A study conducted on pharmacy students in the USA found a decrease in the students' financial and social well-being scores as well as an increase in the percentage of students struggling and suffering during the transition period from COVID-19 to COVID-20 [29].

At the beginning of the global pandemic, Qatar University, the main governmental institution in Qatar, suspended all classes until further notice as part of preventative measures to reduce the spread of the coronavirus and announced the educational process will continue virtually. The impact of this emergency shift to remote teaching increased faculty and students’ academic demands through emergency curriculum changes, financial burdens due to lockdown, and intensified fear of the pandemic itself adversely affecting students’ academic performance, mental health and well-being [30]. Students at Qatar University health colleges are a heterogeneous group from diverse ethnic backgrounds with a large number of non-Qatari students reflecting the population structure with respect to nationality (non-Qatari populations represent 88.4% of the population in Qatar) [31]. Limited studies have assessed the link between anxiety and burnout or burnout and empathy, and even fewer studies have focused on health profession students. Moreover, these studies focused on health profession students from uni-professional perspective and not collectively. Furthermore, to our knowledge, no studies have assessed the relationship between anxiety and burnout and the relationship between burnout and empathy among health profession students using validated instruments. Therefore, this study aims to estimate the prevalence of burnout and assess the relationship between anxiety and burnout and the relationship between burnout and empathy among health profession students during the COVID-19 pandemic. This study is the quantitative phase of a larger research project looking at healthcare students’ wellbeing during the pandemic. The other phase explored health profession students’ coping mechanisms and their experiences of resilience and burnout during the unprecedented COVID-19 pandemic using the Coping Reservoir Model domains to identify depleting and replenishing inputs and examine student resilience and burnout.

## Methods

### Study design, setting and population

An online cross-sectional study was conducted among Qatar University's four health colleges students. These students comprised those from the College of Medicine (CMED), College of Pharmacy (CPH), College of Dental Medicine (CDM) and the College of Health Sciences (CHS). The CHS includes the departments of Public Health, Biomedical Sciences, Human Nutrition, and Physical Therapy and Rehabilitation Science. These four health colleges are under the umbrella of QU Health which was launched in 2017 guided by Qatar’s National Vision 2030 to form an integration between the health colleges (dental, medicine, pharmacy, and health sciences) who work together to prepare competent health profession graduates capable of improving the health sector in Qatar [32].

### Procedure and data collection

Email invitations to participate in the study were sent to all undergraduate and graduate students in QU Health in October 2020 to complete the online survey using SurveyMonkey®. The survey was open from October 2020 until December 2020 and was conducted in English. The survey consisted of five sections: (1) participant’s sociodemographic data; (2) COVID-19 related questions; (3) Generalized Anxiety Disorder 7-item scale (GAD-7); (4) Maslach Burnout Inventory-General Students Survey (MBI-GS(S)); and (5) Interpersonal Reactivity Index (IRI).

Sociodemographic data included gender, age, college and department, year of study (year 1 to year 6), marital status (single, married), employment status (unemployed, part-time or full-time employed), degree level (undergraduate or graduate), nationality (Qatari or Non-Qatari), rating of physical and mental health (poor, fair, good, very good, or excellent), number of courses dropped in Spring semester 2020, and the grade point average (GPA) for Spring 2020 and Fall 2019 semesters.

Using a 5-point Likert scale ranging from “not concerned at all” to “extremely concerned”; students were asked to rate COVID-19 related questions pertaining to the level of concern about personal safety and security; fear of contracting COVID-19; concerns about loved ones contracting COVID-19; number of new COVID-19 cases; and COVID-19 pandemic duration. The extent of following hygiene and social distancing recommendations was measured on a 4-point Likert scale ranging from “not at all” to “very closely”.

Students’ burnout was measured using the MBI-GS(S). MBI scale is a validated and reliable instrument that was introduced in 1981 and is the gold standard for measuring burnout [1]. There are different MBI scales depending on the population studied. MBI-GS (S) is specified for college and university students and has been used in previous studies on health profession students [20, 33], and was found to be a valid and a reliable instrument to measure burnout level among college students [4, 34]. In MBI-GS (S) scale, students rate how frequently they experienced feelings related to burnout using 16 statements measured on a scale of 0 (Never) to 6 (Everyday). The MBI-GS (S) instrument is divided into 3 subscales including exhaustion (MBI-EX), cynicism (MBI-CY), and professional efficacy (MBII-PE) [6].

Generalized Anxiety Disorder 7-item scale (GAD-7) was used to measure generalized anxiety symptoms severity. GAD-7 is a reliable and validated instrument that is used widely as a screening tool for anxiety disorders [35, 36]. A cut-off score of 8 on the GAD-7 was found to have 92% and 76% of sensitivity and specificity for diagnosis of GAD, respectively [37, 38]. The GAD-7 includes 7 items in which respondents answer based on how frequently they experience GAD symptoms using a 4-item Likert scale ranging from 0 (not at all) to 3 (almost every day) [35].

Empathy was measured using the Interpersonal Reactivity Index (IRI) [39]. IRI has been widely used in clinical and research settings that involve health profession students [12, 40, 41]. The IRI assesses four different aspects of empathy including perspective talking, fantasy, empathic concerns, and personal distress. Perspective talking is the tendency of individuals to adopt the psychological point of view of other people whereas fantasy is the tendency to imagine the feelings and actions of fictional characters. Empathic concern describes the feeling of concern or empathy toward others and personal distress measures the feeling of personal anxiety and unease for the individual's self. The IRI contains 28-items answered on a 5-point Likert scale ranging from (does not describe me well) to (describes me very well).

### Statistical analysis

Descriptive statistics were used to summarize characteristics of participants and COVID-19 related variables using mean with standard deviation (SD) for continuous variables and numbers with percentages for categorical variables. For MBI-GS (S), the scores were reported as means for each MBI subscale including MBI-EX, MBI-CY, and MBI-PE. The MBI does not have any cut-off points as it did not show any diagnostic validity. GAD-7 score was categorized into mild (0–5), moderate (6–10) and severe (11–15) [35] Multivariable linear regression was used to assess the relationship between GAD symptoms severity, students’ characteristics, and COVID-19 related variables with MBI sub-scales. In addition, multivariable linear regression was used to assess the relationship between MBI subscales’ scores and the IRI subscales’ scores. Two-sided inferential statistical tests were used and a P-value less than 0.05 was considered statistically significant. Data analysis was performed using the Statistical Package for Social Sciences, version 26 (IBM SPSS® statistics for Windows, IBM Corp, Armonk, New York, USA).

## Results

### Summary of characteristics of participants and COVID-19 related variables

Of the 1268 eligible students, 272 (21.45%) completed the online survey. As shown in Table 1, the mean age was 21.4 years (SD = 3.1). Most participants were females (92.6%), non-Qatari (71.3%), undergraduates (88.6%), single (93.8%), and unemployed (79.8%). The majority of respondents were pharmacy students (36.4%) and medical students (24.6%). The mean and SD of GPA in Fall 2019 and Spring semesters were (3.42, SD = 0.50) and (3.45, SD = 0.49), respectively. Table 2 summarises COVID-19 related variables. The majority of students were very/ extremely concerned regarding COVID-19 duration (69.9%), the number of new cases (61.8%), personal safety and security (54.4%), personally contracting the COVID-19 (44.5%) and their loved ones contracting COVID-19 (79.4%).

**Table 1:** Characteristics of participants

Variable	Mean (SD)	Number (%)
Age, mean (SD)	21.4 (3.1)	
Gender		
Male		20 (7.4)
Female		252 (92.6)
Program		
Biomedical		27 (9.9)
Dental medicine		12 (4.4)
General health sciences		16 (5.9)
Medicine		67 (24.6)
Nutrition		23 (8.5)
Pharmacy		99 (36.4)
Physical therapy		8 (2.9)
Public health		20 (7.4)
Level		
Undergraduate		241 (88.6)
Graduate		31 (11.4)
Year of study		
Year 1		95 (34.9)
Year 2		63 (23.2)
Year 3		48 (17.6)
Year 4		33 (12.1)
Year 5		22 (8.1)
Year 6		11 (4.0)
Nationality		
Qatari		78 (28.7)
Non-Qatari		194 (71.3)
Marital status		
Single		255 (93.8)
Married		17 (6.3)
Employment status		
Unemployed		217 (79.8)
Part-time		25 (9.2)
Full-time		30 (11.0)
Physical health rating		
Poor		41 (15.1)
Fair		51 (18.8)
Good		89 (32.7)
Very good		56 (20.6)
Excellent		35 (12.9)
Psychological and emotional well-being rating		
Poor		73 (25.8)
Fair		100 (36.8)
Good		67 (24.6)
Very good		32 (11.8)
Excellent		0 (0)
Number of courses dropped, mean (SD)	0.09 (0.50)	
GPA in Fall semester 2019, mean (SD)	3.42 (0.50)	
GPA in Spring semester 2020, mean (SD)	3.45 (0.49)	

*SD* standard deviation, *GPA* grade point average

**Table 2:** Summary of COVID-19 related variables

Variable	Number (%)
Most trusted sources	
Government officials	250 (91.9)
Public health agencies	191 (70.2)
University officials	56 (20.6)
National/international media	42 (15.4%)
Local media	15 (5.5%)
Social networking sites	38 (14%)
Discussion forums	4 (1.5%)
Family and friends	26 (9.6%)
Other	15 (5.5%)
Extent of following hygiene recommendations	
Not at all	2 (0.7%)
Not closely	11 (4%)
Somewhat closely	93 (34.2%)
Very closely	164 (60.3%)
Concern regarding COVID19 duration	
Not concerned at all	9 (3.3%)
Slightly concerned	27 (9.9%)
Moderately concerned	45 (16.5%)
Very concerned	93 (34.2%)
Extremely concerned	97 (35.7%)
Extent of following social distancing recommendations	
Not at all	3 (1.1%)
Not closely	22 (8.1%)
Somewhat closely	115 (42.3%)
Very closely	129 (47.4%)
Concern about number of new cases	
Not concerned at all	14 (5.1%)
Slightly concerned	29 (10.7%)
Moderately concerned	60 (22.1%)
Very concerned	80 (29.4%)
Extremely concerned	88 (32.4%)
Concern about personal safety and security	
Not concerned at all	13 (4.8%)
Slightly concerned	43 (15.8%)
Moderately concerned	66 (24.3%)
Very concerned	80 (29.4%)
Extremely concerned	68 (25%)
Concern about personally contracting COVID19	
Not concerned at all	23 (8.5%)
Slightly concerned	47 (17.3%)
Moderately concerned	80 (29.4%)
Very concerned	64 (23.5%)
Extremely concerned	57 (21%)
Concern about loved ones contracting COVID19	
Not concerned at all	6 (2.2%)
Slightly concerned	15 (5.5%)
Moderately concerned	34 (12.5%)
Very concerned	64 (23.5%)
Extremely concerned	152 (55.9%)

### Burnout scores

The means (SDs) of BMI-EX, MBI-CY, and MBI-PE were 4.07 (1.56), 2.63 (1.53), and 3.97 (1.22), respectively.

### Factors associated with burnout

The main factors independently associated with MBI-EX were GAD symptoms severity, year of study, marital status, physical health rating, course withdrawal status, and the extent of following physical distancing measures. Students with severe, moderate, or mild GAD symptoms had higher MBI-EX scores than students with minimal GAD symptoms severity by 0.81 (95% CI 0.32, 1.30), 1.12 (0.59, 1.65), and 1.66 (1.11, 2.21), respectively. As compared to students in year 1, students in years 2, 3, 4, and 6 had higher MBI-EX scores ranging between 0.60 (0.07, 1.13) for year 4 students and 0.91 (0.09, 1.73) for year 6 students. However, this was not statistically significant for year 5 students (Table 3). Married students and those who had withdrawn from 1 or more courses had lower MBI-EX scores than single students and students who elected not to withdraw from a course by 0.84 (0.19, 1.49) and 0.69 (0.04,1.34), respectively. In addition, students who reported following physical distancing measures “very closely” and “not very closely” had higher MBI-EX scores by 1.53 (0.01, 3.06) and 1.83 (0.26, 3.40) than those in the “not at all” category.

**Table 3:** Factors associated with BMI-EX scores

Variable	B (95% CI)	SE	*P* value
GAD severity			
Minimal	Ref		
Mild	0.81 (0.32, 1.30)	0.25	0.001
Moderate	1.12 (0.59, 1.65)	0.27	< 0.001
Severe	1.66 (1.11, 2.21)	0.28	< 0.001
Year of study			
Year 1	Ref		
Year 2	0.82 (0.39, 1.25)	0.22	< 0.001
Year 3	0.63 (0.16, 1.10)	0.24	0.009
Year 4	0.60 (0.07, 1.13)	0.27	0.026
Year 5	0.39 (− 0.22, 1.00)	0.31	0.214
Year 6	0.91 (0.09, 1.73)	0.42	0.028
Marital status			
Single	Ref		
Married	− 0.84 (− 0.19, 1.49)	0.33	0.012
Physical health rating			
Poor	Ref		
Fair	− 0.09 (− 0.64, 0.46)	0.28	0.749
Good	− 0.24 (− 0.75, 0.27)	0.26	0.363
Very good	− 0.76 (− 1.31, − 0.21)	0.28	0.007
Excellent	− 0.14 (− 0.77, 0.49)	0.32	0.667
Course withdrawal			
No	Ref		
Yes	− 0.69 (− 0.04, 1.34)	0.33	0.038
Extent of following physical distancing measures			
Not at all	Ref		
Not very closely	1.83 (0.26, 3.40)	0.80	0.023
Somewhat closely	1.35 (− 0.18, 2.88)	0.78	0.082
Very closely	1.53 (0.01, 3.06)	0.78	0.049

*GAD* generalized anxiety disorder, *B* regression coefficient, *CI* confidence interval, *SE* standard error

The main factors independently associated with MBI-CY were GAD symptoms severity, gender, year of study, nationality, and physical health rating. Students with severe and moderate GAD symptoms had higher MBI-CY scores than students with minimal GAD symptoms severity by 1.10 (0.53, 1.67) and 0.56 (0.01, 1.11), respectively (Table 4). As compared to students in year 1, students in years 2, 3 and 5 had higher MBI-CY scores ranging between 0.67 (0.18, 1.16) for year 3 students and 0.94 (0.31, 1.57) for year 5 students. However, this was not statistically significant for years 4 and 6 students (Table 4). Female and non-Qatari students had lower MBI-CY scores than male and Qatari students by 0.93 (0.28, 1.58) and 0.41 (0.04, 0.78), respectively.

**Table 4:** Factors associated with cynicism domain

Variable	B (95% CI)	SE	*P* value
GAD severity			
Minimal	Ref		
Mild	0.43 (− 0.08, 0.94)	0.26	0.092
Moderate	0.56 (0.01, 1.11)	0.28	0.045
Severe	1.10 (0.53, 1.67)	0.29	< 0.001
Gender			
Male	Ref		
Female	− 0.93 (− 1.58, − 0.28)	0.33	0.005
Year of study			
Year 1	Ref		
Year 2	0.76 (0.33, 1.19)	0.22	0.001
Year 3	0.67 (0.18, 1.16)	0.25	0.007
Year 4	0.48 (− 0.07, 1.03)	0.28	0.083
Year 5	0.94 (0.31, 1.57)	0.32	0.003
Year 6	0.75 (− 0.09, 1.59)	0.43	0.081
Nationality			
Qatari	Ref		
Other	− 0.41 (− 0.78, 0.04)	0.19	0.030
Physical health rating			
Poor	Ref		
Fair	0.01 (− 0.56, 0.58)	0.29	0.965
Good	− 0.46 (− 0.99, 0.07)	0.27	0.082
Very good	− 0.70 (− 1.27, − 0.13)	0.29	0.015
Excellent	− 0.45 (− 1.10, 0.20)	0.33	0.166

*GAD* generalized anxiety disorder, *B* regression coefficient, *CI* confidence interval, *SE* standard error

The main factors independently associated with MBI-PE were GAD symptoms severity, study program, extent of following physical distancing measures, and sense of personal safety and security. Students with moderate GAD symptoms had lower MBI-PE scores than students with minimal GAD symptoms severity by 0.72 (0.27, 1.17). However, this was not statistically significant for students with severe or mild GAD symptoms severity (Table 5).

**Table 5:** Factors associated with professional efficacy domain

Variable	B (95% CI)	SE	*P* value
GAD severity			
Minimal	Ref		
Mild	− 0.24 (− 0.71, 0.23)	0.24	0.279
Moderate	− 0.72 (− 1.17, − 0.27)	0.23	0.002
Severe	− 0.36 (− 0.79, 0.07)	0.22	0.134
Program			
Biomedical	Ref		
Dental medicine	0.47 (− 0.35, 1.29)	0.42	0.265
General health sciences	1.01 (0.28, 1.74)	0.37	0.007
Medicine	− 0.20 (− 0.71, 0.31)	0.26	0.446
Nutrition	0.28 (− 0.37, 0.93)	0.33	0.391
Pharmacy	0.29 (− 0.20, 0.78)	0.25	0.248
Physical therapy	0.58 (− 0.32, 1.48)	0.46	0.203
Public health	0.93 (0.26, 1.60)	0.34	0.006
Extent of following physical distancing measures			
Not at all	Ref		
Not very closely	2.71 (1.34, 4.08)	0.70	< 0.001
Somewhat closely	2.57 (1.22, 3.92)	0.69	< 0.001
Very closely	2.57 (1.24, 3.90)	0.68	< 0.001
Sense of personal safety and security			
Not concerned at all	Ref		
Slightly concerned	0.38 (− 0.31, 1.07)	0.35	0.281
Moderately concerned	0.74 (0.07, 1.41)	0.34	0.030
Very concerned	0.78 (0.11, 1.45)	0.34	0.021
Extremely concerned	1.09 (0.40, 1.78)	0.35	0.002

*GAD* generalized anxiety disorder, *B* regression coefficient, *CI* confidence interval, *SE* standard error

### The relationship between burnout and empathy

MBI-EX was significantly and independently associated with empathetic concern and personal distress subscales of empathy. Increase in MBI-EX by 1 score was associated with increase in empathetic concern and personal distress scores by 0.76 (0.41, 1.11) and 0.77 (0.42, 1.12), respectively. However, no statistically significant associations were found between MBI-EX and perspective talking and fantasy subscales of empathy (Table 6). MBI-CY was associated only with personal distress. Increase in MBI-CY by 1 score was associated with increase in personal distress score by 0.36 (0.01, 0.71). In addition, MBI-PE was associated only with perspective talking and empathetic concern subscales of empathy. Increase in MBI-PE by 1 score was associated with increase in perspective talking and empathetic concern scores by 1.14 (0.71, 1.57) and 0.85 (0.44, 1.16), respectively.

**Table 6:** Adjusted relationship between burnout domains and empathy domains

MBI domains	Perspective taking			Fantasy			Empathic concern			Personal distress		
	B (95% CI)	SE	*P* value	B (95% CI)	SE	*P* value	B (95% CI)	SE	*P* value	B	SE	*P* value
MBI-EX	0.003 (− 0.37, 0.38)	0.19	0.989	0.19	0.24	0.432	0.76 (0.41, 1.11)	0.18	< 0.001	0.77 (0.42, 1.12)	0.18	< 0.001
MBI-CY	− 0.25 (− 0.62, 0.12)	0.19	0.191	− 0.25 (− 0.66, 0.28)	0.24	0.299	− 0.22 (− 0.59, 0.15)	0.19	0.253	0.36 (0.01, 0.71)	0.18	0.045
MBI-PE	1.14 (0.71, 1.57)	0.22	< 0.001	0.59 (0.02, 1.16)	0.29	0.039	0.85 (0.44, 1.26)	0.21	< 0.001	− 0.21 (− 0.62, 0.20)	0.21	0.311

*BMI* Maslach Burnout Inventor, *EX* exhaustion, *CY* cynicism, *PE* professional efficacy, *B* regression coefficient, *CI* confidence interval, *SE* standard error, *SD* standard deviation, *GPA* grade point average

## Discussion

The COVID-19 pandemic has disrupted many aspects of life, especially education. This study aimed to assess how COVID-19 pandemic affected the students’ life in regard to the level of burnout and the relationship between anxiety and burnout, and burnout and empathy amongst QU Health students. In contrast to many of literature findings, gender was not associated with higher levels of burnout [42, 43]. QU Health majority of students are female, and some of its colleges are exclusively for females, such as the College of Health Sciences and College of Pharmacy. Recently, the latter began enrolling male students. Our study found that anxiety is a strong predictor for burnout. The study data suggested that all levels of anxiety symptoms (mild, moderate, severe) were statistically significant (minimal anxiety level is the reference) when applied into the multiple linear regression model with each MBI subscale (emotional exhaustion, cynicism, professional efficacy). Yet the most significant association was between anxiety and the emotional exhaustion and cynicism subscales of burnout. Similar findings were also reported in previous studies where a positive association was found between symptoms of anxiety and burnout [23, 44, 45]. Moreover, A cross-sectional study conducted among medical students and residents in Nepal assessed depression, anxiety, and burnout, and found that anxiety is a significant positive predictor of burnout. t (OR 2.37; 95% CI 1.51–3.75) [23]. A second cross-sectional study was conducted amongst health professionals in China showed that emotional exhaustion and cynicism were significantly correlated to symptoms of anxiety, mean (SD) = 10.1 (6.5), 5.7 (5.2), 37.7 (9.7) respectively; *p* < 0.01 (two-tailed) for both EE and CY [21].

This study found that burnout was positively associated with empathy which is contradictory to the literature findings that suggest that burnout reduces empathy of students [11–13, 19, 46, 47]. It is worth noting that all these studies reported in the literature were before the COVID-19 pandemic. This might explain how the study results contradict with the results of these studies. A cross-sectional study conducted in Pakistan amongst dental students has addressed how COVID-19 affects the level of empathy of the students [48]; which found that significant increase in empathy levels pre- and post-COVID-19. Although the students had increased burnout levels, empathy was increased which highlights how COVID-19 pandemic affected this possible behavioural change.

Burnout has a negative influence on the students’ quality of life, and if persisted, it can lead to detrimental consequences on the students’ mental and physical health as well as their careers [49]. As a result, assessment of students' burnout is crucial in order to create a healthy academic environment and foster their resilience. Developing protective factors can also aid students in becoming more resilient [50]. These protective factors include coping mechanisms, peer and family support, social connectedness, mentorship, and intellectual stimulation [50]. Other proposed strategies to increase students’ resilience and well-being include cognitive-behavioural counselling services, time management training, emotional teaching, exercise interventions, and mindfulness based therapy [49, 51]. Educational institutions must frequently assess their students’ wellbeing and provide a safe environment for them in order to discuss the challenges that they face and to work together to find appropriate solutions.

This study was the first to assess and correlate burnout, anxiety, and empathy amongst students and to assess it amongst health profession students. Another strength of this study is the use of multiple logistics regression which has high statistical power in determining the presence of associations/relationships. The main limitation of this study was the low response rate (21.45%) which could be due to the length of the survey. Another potential limitation is the possibility of social desirability bias which can occur in any cross-sectional study. In addition, the study was conductedat a single institution which limits its generalizability to other academic health institutions in the country such as Weill Cornell Medicine- Qatar, University of Calgary- Qatar and University of Doha for Science and Technology Another limitation is that most participants were female students (93%). Although this reflects the current proportion of female students in colleges within QU Health, the findings of this study may not be generalized to male students. However, consistent with the findings of other studies, female gender has been shown to be a predictor of burnout [52, 53] and a significant gender difference in empathy was shown in some studies [54]. Unfortunately, in the absence of prior studies, there was no comparative data for burnout in this cohort of students prior to the pandemic, as it would have been interesting to compare it to the previous findings to further showcase the effect of the pandemic on our results in terms of burnout, anxiety and empathy and their associations with each other. However, this study captures a temporal moment that is unlikely to be captured again i.e. during a pandemic. Future research may include a larger scale study to include all health profession students in Qatar, or a different population of students (non-health profession students). In addition, research on burnout can be conducted on the university staff and professors to explore their experiences of the pandemic and how COVID-19 affected them and their delivery of education. As this study highlighted a possible behavioural change secondary to the effect of COVID-19 pandemic, further studies can be conducted to explore empathy and other psychological changes in students and the general population amid the pandemic.

## Conclusion

Findings from this study demonstrated relationships between health profession students’ burnout, anxiety, and empathy. Anxiety was found to be a strong predictor for burnout and burnout was positively associated with empathy. These findings might have an impact on the development of curriculum interventions to enhance student well-being. More burnout awareness and management programs that cater to the specific needs of health profession students are needed to help reduce the prevalence and consequences of burnout, as well as, to increase the availability of mental health services for students. Furthermore, it may have implications for future educational interventions during times of crisis or how this can be used to improve student experiences in normal times.

## Data Availability

The datasets used and/or analysed during the current study available from the corresponding author on reasonable request.
